# Relationship between the Polymer Blend Using Chitosan, Polyethylene Glycol, Polyvinyl Alcohol, Polyvinylpyrrolidone, and Antimicrobial Activities against *Staphylococcus aureus*

**DOI:** 10.3390/pharmaceutics15102453

**Published:** 2023-10-13

**Authors:** Linh Doan, Khoa Tran

**Affiliations:** 1 School of Chemical and Environmental Engineering, International University—Vietnam National University, Ho Chi Minh City 70000, Vietnam; 2 Nanomaterials Engineering Research & Development (NERD) Laboratory, International University—Vietnam National University, Ho Chi Minh City 70000, Vietnam

**Keywords:** antimicrobial resistance, polymer blends, chitosan, optimization, Taguchi, antibacterial and antifungal, wound healing

## Abstract

The findings from Pareto charts, main effect plots, and interaction plots demonstrate the importance of polymer concentration. Increasing concentration improves the inhibition percentage and decreases the MIC_50_. However, the primary factor that influences these changes is chitosan (CS). Additionally, the interaction between CS and PVP, along with other polymers, plays a crucial role in achieving better antimicrobial effects. These results enhance our understanding of the antimicrobial properties of the studied polymers and offer valuable insights for developing effective antimicrobial formulations. The MIC_50_ value of M1–M16 was at a polymer percentage of 12.5%. At 12.5% polymer percentage, with the limits of [PVA], [PEG], and [PVP] being 0.002–0.004 g/mL and [CS] being 0.001–0.002 g/mL, using the 2-level full factorial method, the inhibition percentage is equal to 174.1 − 27,812 PVA − 18,561 PVP − 25,960 PEG − 38,752 CS + 9,263,047 PVA*PVP + 10,430,763 PVA*PEG + 15,397,157 PVA*CS + 7,088,313 PVP*PEG + 7,841,221 PVP*CS + 14,228,046 PEG*CS − 3,367,292,860 PVA*PVP*PEG − 5,671,998,721 PVA*PVP*CS − 6,619,041,275 PVA*PEG*CS − 3,917,095,529 PVP*PEG*CS + 2,273,661,969,470 PVA*PVP*PEG*CS. Theoretically, the most economical concentrations of PVA, PVP, PEG, and CS are 0.002, 0.002, 0.002, and 0.001 mg/mL at a concentration of 12.5% to reach an inhibition percentage of 99.162%, which coincides with the MBC value.

## 1. Introduction

Microorganisms can cause harmful effects on human health, especially in dental products, food packaging and storage, water purification systems, and household sanitation [1]. One of the most harmful microorganisms is *Staphylococcus aureus* (SA). SA can infect individuals in various settings, including both the community and healthcare facilities. It is responsible for causing a wide range of infections in humans, such as bloodstream infections, heart valve infections, skin and soft tissue infections, bone and joint infections, and infections acquired in hospitals [2,3]. One of the most common places to find SA is in dry parts of the skin with atopic dermatitis [4,5,6,7]. Currently, SA can be killed by antibiotics or germicides, which can cause irritation and antimicrobial resistance (AMR) [7]. However, the most concerning problem with this method is AMR. AMR has become a critical global health issue in recent times. SA is a type of bacteria that has developed resistance to antibiotics and is causing a considerable public health problem [2,8]. Hence, the use of other methods that do not cause AMR but still have the ability to kill SA is necessary. One of the methods is developing an antimicrobial polymer acting as a protective barrier in dermal injuries, which can be in gels or patches for topical applications including cosmetics or drug delivery and require a balance between physical strength and antimicrobial activity [9,10,11].

Antimicrobial polymers, which can inhibit or destroy the growth of microorganisms, can be found in nature or in synthesis. The ability of polymers to fight against microorganisms depends on factors such as the functional groups present on their surface, their surface charge, and their molecular weight [9,12,13,14,15]. Bacterial cell walls have a negative charge, allowing them to attract and bond with positively charged polymers [9,16,17]. The bactericidal effect of polymers, like chitosan (CS), one of the most common antimicrobial polymers and highly effective against Gram-negative bacteria, can be found in abundance naturally in shrimp waste [1,18]. It involves a two-step process. Firstly, the polymer adheres to the bacterial cell wall and then penetrates through it. Secondly, the polymer disrupts the cell membrane, causing the leakage of internal cell contents and ultimately leading to cell death [9,19]. Because CS is more effective against Gram negative bacteria [1], and because of the danger of SA, the antibacterial activity of the polymer blend was carried out on Gram-positive bacteria—SA, which can be isolated from a human corneal ulcer [20].

Recently, CS has blended with synthetic polymers, which can be considered a great method to design new polymeric materials with many attractive properties suitable for many application requirements that cannot be achieved by a single polymer (i.e., biodegradable, improved mechanical properties) [21,22,23,24,25,26,27]. One of the polymers that can be blended with CS is polyvinyl alcohol (PVA), a non-toxic, water-soluble, biodegradable, biocompatible, widely-used, and outstanding physical and chemical polymer that can be used in various applications such as membrane preparations, drug delivery, recycling polymers, and textiles [21,28,29,30,31,32]. However, because of its poor stability in water properties, PVA should be blended with another polymer such as polyvinyl pyrrolidone (PVP), which is a hydrophilic, low-cytotoxicity, biocompatible polymer widely used in biomedical applications such as wound healing matrix, antifungal agents, antiseptics, and transdermal drug delivery [33,34,35]. To further improve mechanical properties, nontoxicity, noncarcinogenic effect, and bioadhesive properties, polyethylene glycol (PEG) can be used to blend with PVA, which forms strong interactions due to hydrogen bond formation [36,37,38]. Hence, many researchers combined at least two of these mentioned polymers—PVA, PVP, PEG, and CS—to create new antimicrobial agents with various applications [39,40,41,42,43,44,45,46,47,48,49]. Additionally, these polymers are capable of degrading naturally, compatible with biological systems, have the ability to form films, and have been previously documented for their use in topical applications [9,50,51]. However, most research does not provide an insight into the relationship between the polymer blend composition and the antimicrobial activity.

Hence, in this work, the relationship between the ratio of PVA/PVP/PEG/CS and the antimicrobial activities was determined. The mass fractions of PVA, PVP, and PEG selected in this study follow US Food and Drug Administration (FDA) approval [52,53,54]. More importantly, this research is mainly focused on using statistical analysis such as Pareto charts, main effect plots, and interaction plots to determine if the polymer composition affects the antimicrobial activity. Moreover, from these statistical analyses, the inhibition percentage can be predicted based on the concentration of individual polymers (CS, PEG, PVP, and PVA). Therefore, this work should be used as the basis for further study on the inhibition mechanism and the interaction between polymers.

## 2. Experimental Methods

Materials: polyvinyl alcohol (PVA) and polyethylene glycol-1000 (PEG) were purchased from Xilong Scientific Co., Ltd. (Shantou, China). Chitosan (CS) and polyvinylpyrrolidone (PVP K30) were purchased from Shanghai Zhanyun Chemical Co., Ltd. (Shanghai, China). Glacial acetic acid (AA) was purchased from RCI Labscan (Bangkok, Thailand). All materials were used as received.

Synthesis of CS/PEG/PVA/PVP: To make a polymer blend, polymer stock solutions had to be made. The stock polymer was synthesized by adding dried 1 g PEG, 1 g PVA, and 1 g PVP in 50 mL DI water individually. Meanwhile, 0.5 g of CS was added to 48.5 mL of DI water and 1.5 mL of AA. The polymer stocks were stirred and heated at 80 °C for 1 h, or until the polymers were completely dissolved in water. Then, each stock polymer was mixed with additional DI at the volume shown in Table 1 and Table 2 in 15 mL plastic falcon tubes. Then, the mixture was shaken in an orbital incubator at 35 °C and 150 rpm for 20 h. All the experiments were repeated 3 times.

### Evaluating the Antibacterial Activities of Polymer Blends

*Staphylococcus aureus* strain ATCC 29523 was obtained from the School of Biotechnology, International University—VNU HCM and grown in Mueller Hinton Broth (MHB) (Himedia Laboratories, India) for 24 h at 37 °C under aerobic conditions. Gram staining, followed by microscopic observation, was performed for confirmation.

The minimum inhibitory concentration (MIC) test was assessed using the modified microdilution method [55,56]. Briefly, bacterial broth culture was standardized to 5 × 10^7^ CFU/mL by optical density (0.05 at OD600 nm) in autoclaved MHB using a DR6000 UV VIS Spectrophotometer (Hach, CO, USA). The growth inhibition was established in sterile flat-bottom 96-well plates (Biologix, MO, USA). The polymer blends were prepared as described in the synthesis of PVA/PVP/PEG/CS section to 1.953 µg/mL for *S. aureus* in distilled water via two-fold serial dilution. Each well of the sterile 96-well plate was filled with 50 µL of standardized bacterial suspension (*S. aureus*) and 50 µL of different concentrations of polymer blends.

The 96-well plates were incubated at 37 °C for 24 h in aerobic conditions for *S. aureus* using a Memmert Model 30-1060 incubator (Memmert GmbH, Schwabach, Germany). Optical density at 600 nm (OD600) was measured before the incubation (0 h post-inoculation, t_0_). The second OD was measured post-inoculation. Optical density was read by a Synergy HT multimode plate reader (BioTek, Winooski, VT, USA).

The percentage of inhibition was calculated using the formula shown in Equation (1):

(1) $$\%\;\mathrm{inhibition}=\left(1-\left(\frac{{\mathrm{OD}}_{600/t}-{\mathrm{OD}}_{600/t_{0}}}{{\mathrm{OD}}_{\left(-\right)600/t}-{\mathrm{OD}}_{\left(-\right)600/t_{0}}}\right)\right)\times 100,$$

with:

OD_600/t_ = optical density (600 nm) of the test well at 24 h or 72 h post-inoculation;
OD_600/t_0__ = optical density (600 nm) of the test well at 0 h post-inoculation;
OD_(−)600/t_ = optical density (600 nm) of the negative control well at 24 h or 72 h post-inoculation;
OD_(−)600/t_0__ = optical density (600 nm) of the negative control well at 0 h post-inoculation.

Minimum Bactericidal Concentration (MBC) was established for the best-performing polymer blend by plating the MIC concentration plus two more concentrated concentrations on MH agar (Himedia Laboratories, India) plates and counting the colony growth after 24 h of incubation at 35 °C.

Data analysis: Taguchi’s orthogonal array table was prepared with four chosen parameters. In this experiment, the four parameters and the levels used were four types of polymers: PVA, PVP, PEG, and CS. Two concentration levels were selected: 0.002 g/mL and 0.004 g/mL. The polymers were blended at different ratios and tested for their antimicrobial activities. After testing the antimicrobial activities, the minimal inhibition concentration (MIC) and inhibition percentage at various dilutions were analyzed to determine the relationship between each individual polymer and antimicrobial activities. The Signal-to-Noise ratio is utilized to determine the optimal levels of factors. It is a performance measure created by Taguchi that identifies the parameter levels that maximize this ratio. In this context, “signal” represents the desired quality characteristic, while “noise” indicates the variability (measured by variance) of the characteristics. The specific equation for the Signal-to-Noise ratio depends on the criteria for optimizing the quality characteristic. In this experiment, due to optimizing the antimicrobial activities of the polymers, the standard Signal-to-Noise ratios used were “smallest-is-best” for MIC_60_ (minimal inhibition concentration when at least 60% of the bacteria was inhibited), which was shown in Equation (2) [57,58] and “larger-is-better” for inhibition percentage (Equation (3)) [59]:

(2) $$\frac{S}{N}=-10\times \log\left(\sum \left(Y^{2}\right)/n\right),$$

(3) $$\frac{S}{N}=-10\times \log\left(\sum \left(\frac{1}{Y^{2}}\right)/n\right),$$

where Y is the response for the given factor level combination and n = number of responses in the factor level combination.

On the other hand, as shown in Table 2, each experimental factor has only two levels, and the experimental runs include all combinations of these factor levels. Hence, a 2-level full factorial design was used to determine how each individual polymer and the interaction between polymers affect the antimicrobial activities. Moreover, linear regression was also used to determine these relationships. To determine whether the experimental data fit the regression, the chi-square test was tabulated as shown in Equation (4) [60]:

(4) $$\chi^{2}=\sum_{i=1}^{m}\frac{{\left(Q_{e,\exp}-Q_{e,\mathrm{calc}}\right)}^{2}}{Q_{e,\exp}},$$

The software to calculate Taguchi and 2-level factorial design, as well as linear regression, was Minitab-17 and Microsoft Excel 365 v.2309.

## 3. Results and Discussion

By using different ratios of polymers in the MIC experiments, the inhibition percentage can be calculated using Equation (1) and plotted against the polymer percentage, as shown in Figure S1.

As shown in Figure S1, as the polymer percentage decreases, the error bars get larger. This indicates that reliable data can be considered at a polymer percentage of 12.5%. Moreover, at a 50% inhibition percentage or greater, these reliable polymer percentages were present. Hence, the MIC values at 50% inhibition, or MIC_50_, can be seen at the polymer percentage of 12.5% overall. However, when analyzing in detail each polymer percentage for M1–M16, the effects of the polymer blend on antibacterial activities, such as the inhibition percentage at various dilutions and the MIC values, can be portrayed graphically through the Pareto chart (which can only be obtained by the 2-level factorial design method), main effects plots, and interaction plots, which can be obtained by 2-level factorial design methods and Taguchi methods.

The 2-level factorial design method can generate the Pareto charts (as shown in Figures S2 and S3), which illustrate the absolute magnitude of the impacts and include a benchmark line. Any impact that surpasses this benchmark line (vertical red dotted line) is considered statistically significant at the 95% confidence level [61,62].

As shown in Figure S2, the Pareto chart indicates that the MIC_50_ heavily depends on the concentration of CS. As shown in Figure S3, the Pareto chart indicates that the inhibition percentage heavily depends on the concentration of CS. When the polymer percentage was 6.25%, apart from CS and PVA, the interaction of PVP and CS also significantly affected the MIC_50_. However, when the blend has 25% polymers, the inhibition percentage can also be affected by the interaction between PVA and PVP, even though the confidence level is less than 95%. On the other hand, when the blends are not diluted, the inhibition percentage can also be affected by the interaction of PVA and PEG, even though the confidence level is less than 95%. This indicates that each polymer can affect the inhibition percentage as well as the MIC_50_ values, but mainly CS.

To determine which factor affects the response value, the main effect plots should be generated. The main effects plot illustrates the fundamental impact of altering the significant factors. These effects, known as main effects, are represented by lines on the plot. A larger main effect is indicated by a line with a steeper slope compared to the effects contributed by less significant factors. To determine the main effects, the Minitab procedure calculates the difference between the mean response at the low or first level of the factor and the mean response at the high or second level of the factor [63,64].

Hence, the main effect plots on MIC_50_ and inhibition percentage and MIC_50_ for M1–M16 polymer percentage of 100%, 50%, 25%, 12.5%, 6.25%, and 3.125% using 2-level factorial design methods were generated as shown in Figures S4 and S5.

As shown in Figures S4 and S5, each polymer affects the inhibition percentage and MIC_50_. However, CS shows remarkably larger effects compared to PVA, PEG, and PVP. Depending on the polymer percentage, CS can be affected positively or negatively as concentration increases to the inhibition percentage. On the other hand, for MIC_50_, as the concentration of CS increases, the means of MIC_50_ decreases much greater than the increases of PVA, PEG, and PVP. This indicates that the higher the concentration of polymers, the lower the MIC_50_ value. However, as PVP and PEG increase, the MIC_50_ value decreases insignificantly. Safely to say, to optimize the MIC_50_ value, CS and PVA must be increased, while PVP and PEG can be kept at 0.002 g/mL. On the other hand, as the polymer percentage varies, the effects of individual polymers might differ. However, overall, CS is still the main factor affecting the inhibition percentage. This trend can be confirmed by the Taguchi method and linear regression, as shown in Figures S6 and S7.

As shown in Figures S6 and S7, due to the discrepancies in the effects on the inhibition percentage at different polymer percentages, some interactions between the polymers might occur and change the antibacterial activities. To visualize the influence of different factor combinations and determine the most significant factors, interaction plots were created. These plots also consider the interactions between variables and are useful for optimizing operational parameters in systems with multiple variables [64,65]. The plot represents the average response for all possible combinations of settings for the two factors. Hence, utilizing an interaction plot to visually depict how the connection between a specific categorical factor and a continuous response varies based on the value of a second categorical factor this plot illustrates the average values for different levels of one factor along the x-axis, with each level of the other factor represented by a distinct line. By examining the lines, the interactions impact the association between the factors, and the response can be determined. The parallel lines indicate the absence of interactions, while the nonparallel lines indicate the presence of an interaction. The extent of nonparallelism in the lines reflects the strength of the interaction [63]. Hence, as shown in Figures S8 and S9, the interaction plots of the effects of M1–M16 on MIC_50_ and inhibition percentage when the polymer blends with polymer percentages of 100%, 50%, 25%, 12.5%, 6.25%, and 3.125% using 2-level factorial design methods.

As shown in Figures S8 and S9, the most visual interactions between CS-PEG, CS-PVP, PEG-PVP, PVP-PEG, and PVP-CS can be seen. This trend can be confirmed by the Taguchi method, as shown in Figures S10 and S11.

As shown in Figures S2–S11, the utilization of Pareto charts, main effect plots, and interaction plots has shed light on the relationship between polymer concentration and antibacterial activity. The findings indicate that as the concentration of polymers increases, there is a corresponding increase in inhibition percentage and a decrease in MIC_50_. However, it is evident that CS plays a pivotal role in driving these changes, emerging as the primary factor influencing antimicrobial efficacy. Furthermore, the examination of interaction plots revealed that the main interactions within the polymer blends were observed between CS, PVP, and the other polymers. These interactions potentially contribute to enhanced antimicrobial properties and demonstrate the importance of considering the synergistic effects of different polymers in antimicrobial applications.

### 3.1. Predictions between Antibacterial Activities and Polymer Concentration

The MIC experiments showed that the polymer concentration greatly affects the inhibition percentage and the MIC values. These effects can be predicted and regressed using two methods: a 2-level factorial design and linear regression. As shown in Equation (5) and Table 3a,b, the 2-level factorial design shows the relationship between inhibition percentage at various dilution times and the concentration of polymers:

(5) $${\%\mathrm{inhibition}}_{\mathrm{Polymer}\;\mathrm{percentage}}=c_{0}+c_{1}\times \left[A\right]+c_{2}\times \left[B\right]+c_{3}\times \left[C\right]+c_{4}\times \left[D\right]+c_{5}\times \left[\mathrm{AB}\right]\;+c_{6}\times \left[\mathrm{AC}\right]+c_{7}\times \left[\mathrm{AD}\right]+c_{8}\times \left[\mathrm{BC}\right]+c_{9}\times \left[\mathrm{BD}\right]+c_{10}\times \left[\mathrm{CD}\right]+c_{11}\times \left[\mathrm{ABC}\right]\;+c_{12}\times \left[\mathrm{ABD}\right]+c_{13}\times \left[\mathrm{ACD}\right]+c_{14}\times \left[\mathrm{BCD}\right]+c_{15}\times \left[\mathrm{ABCD}\right],$$

where c_0_ to c_14_ are constants shown in Table 3 and [A], [B], [C], and [D] are the concentrations of PVA, PVP, PEG, and CS (g/mL), respectively, and [AB] is the multiplication of the concentrations of [A] and [B].

As shown in Table 3, the $\chi^{2}$ values were quite small and close to zero. Hence, Equation (5) can be used as a prediction tool for the relationship between inhibition percentage and polymer concentration, which were graphically depicted as shown in Figure S13.

As shown in Figure S13, with a polymer percentage higher than 12.5%, the error bars were much smaller compared to a lower polymer percentage. At a polymer percentage of 25%, the inhibition percentage slightly decreases and reaches equilibrium at a polymer percentage of 50% or greater. Additionally, the peak inhibition percentage was at a polymer percentage of 12.5%. Hence, the MIC_50_ of M1–M16 can be at 12.5%. Based on the MIC_50_ value of 12.5%, combining with Figures S5 and S7, the CS still contributes significantly to the inhibition percentage, while PVA, PVP, and PEG contribute insignificantly. Based on Equation (5) and using constants in Table 3b, theoretically, the highest inhibition percentage value at different polymer percentages, the concentration of PVA, PVP, PEG, and CS, can be calculated as shown in Table 3c.

Based on Table 3c, the highest inhibition percentage can be obtained when the polymer percentage were 12.5% and 6.25%. However, due to the small increase in inhibition percentage and the double concentration of each individual polymer, economically, the most optimized polymer percentage is at 12.5%, and the concentration of PVA, PVP, PEG, and CS should be 0.002, 0.002, 0.002, and 0.001 mg/mL. Compared to the experimental data on the inhibition percentage of M8 at 12.5% polymers, the experimental value of the inhibition percentage was 99.168%. Hence, based on Table 3 and Equation (5), the inhibition percentage can be predicted based on the concentration of each individual polymer and the polymer percentage, with some constraints as 0.002 g/mL ≤ [PVA] ≤ 0.004 g/mL, 0.002 g/mL ≤ [PVP] ≤ 0.004 g/mL, 0.002 g/mL ≤ [PEG] ≤ 0.004 g/mL, and 0.001 g/mL ≤ [CS] ≤ 0.002 g/mL. If these constraints were not considered, based on Figures S9 and S11, decreasing the concentration of PVA, PVP, and PEG might affect the inhibition percentage due to some interactions between CS-PVA, CS-PVP, CS-PEG, PEG-PVA, PEG-PVP, PVP-PVA, PVA-PVP, PVA-PEG, and PVP-PEG.

On the other hand, linear regression was used to show the relationship between inhibition percentage at various dilution times and the concentration of polymers, as shown in Equation (6) and Table 4:

(6) $${\%\mathrm{inhibition}}_{\mathrm{Polymer}\;\mathrm{Percentage}}=c_{0}+c_{1}\times \left[A\right]+c_{2}\times \left[B\right]+c_{3}\times \left[C\right]+c_{4}\times \left[D\right].$$

Looking at Table 4, the R^2^ values were not larger than 90, indicating that the linear regression should not be used as a prediction tool for the inhibition percentage based on polymer percentage. Similarly, the relationship between MIC_50_ and the concentration of polymers can be calculated using the 2-level factorial design as shown in Equation (7) and Table 5:

(7) $${\mathrm{MIC}}_{50}=c_{0}+c_{1}\times \left[A\right]+c_{2}\times \left[B\right]+c_{3}\times \left[C\right]+c_{4}\times \left[D\right]+c_{5}\times \left[\mathrm{AB}\right]+c_{6}\times \left[\mathrm{AC}\right]+c_{7}\times \left[\mathrm{AD}\right]+c_{8}\times \left[\mathrm{BC}\right]\;+c_{9}\times \left[\mathrm{BD}\right]+c_{10}\times \left[\mathrm{CD}\right]+c_{11}\times \left[\mathrm{ABC}\right]+c_{12}\times \left[\mathrm{ABD}\right]+c_{13}\times \left[\mathrm{ACD}\right]+c_{14}\times \left[\mathrm{BCD}\right]+c_{15}\times \left[\mathrm{ABCD}\right],$$

where c_0_ to c_14_ are constants shown in Table 5 and [A], [B], [C], and [D] are the concentrations of PVA, PVP, PEG, and CS (g/mL), respectively, and [AB] is the multiplication of the concentrations of [A] and [B].

As shown in Table 5, the $\chi^{2}$ was quite small, indicating that Equation (7) can be used as a prediction tool. On the other hand, linear regression was used to show the relationship between MIC and the concentration of polymers, as shown in Equation (8) and Table 6:

(8) $${\mathrm{MIC}}_{50}=c_{0}+c_{1}\times \left[A\right]+c_{2}\times \left[B\right]+c_{3}\times \left[C\right]+c_{4}\times \left[D\right].$$

As shown in Table 6, the R^2^ values were not greater than 90, indicating that the linear regression or Equation (8) should not be used as a prediction tool. To determine whether the linear regression fitted the ordinary least-square assumptions, residual plots were generated, including the normal probability plot, versus fits, histograms, and versus order. The normal probability plots validate the assumption of a normal distribution for the residuals. This plot allows for an assessment of whether the residuals follow a normal distribution pattern. The patterns that should violate the assumption of normal distribution and should not be seen are the S-curve, inverted S-curve, downward curve, or a few points away from other points.

The residual versus fit plots verify the assumption of constant variance in the residuals. This plot helps ascertain whether the spread of residuals remains consistent across the range of predicted values. If a pattern such as fanning, curvilinear, far-away-from-zero point, or far-away-from-other-points-in-x-direction point might indicate the existence of nonconstant variance, missing higher-order term, outlier, or influential point, respectively.

The histogram of residuals examines the distribution of the differences between observed and predicted values. This will help determine if the data displays any skewness or if there are any outliers present. If the pattern has a long tail in one direction or a bar that is isolated, the pattern might indicate skewness or an outlier, respectively.

The residuals versus order of data plots confirm the assumption of no correlation among the residuals. This plot enables an assessment of whether there is any systematic pattern or relationship between the residuals and their order in the dataset. Hence, if a pattern is spotted, the indication of dependent residuals might occur, and an investigation should be performed. These patterns can be in the shape of a trend, shift, or cycle.

Hence, the residual plots of the linear regression of polymer blend concentration and inhibition percentage at different dilution times and MIC_60_ were generated, as seen in Figures S13 and S14.

As seen in Figures S13 and S14, the residual plots confirmed that the linear regression would not be a good fit to predict the relationship between polymer blend concentration and inhibition percentage at different dilution times and MIC_50_. One of the possible explanations is that the linear regression did not consider the interaction between the polymers. Hence, to predict the relationship, Equations (5) and (7) should be used.

### 3.2. FTIR Characterization

After determining the highest inhibition percentage polymer blend—M8 sample, the individual polymers and M8 sample were analyzed using the FTIR analysis, as shown in Figure S15 and Table 7. Fourier transform infrared (FTIR) spectroscopy was used to characterize the changes in specific functional groups. FTIR spectra were obtained by a FT-IR microscope spectrometer (LUMOS, Bruker, Germany) in the spectral range of 400–4000 cm^−1^.

As shown in Figure S15 and Table 7, the peaks of pure PVA, PVP, PEG, and CS + AA were analyzed. Due to the low concentration of pure polymers in water, the intensity of O-H symmetric stretching was significant. However, the characteristic peaks of each individual polymer, such as the C–H bending vibration of CH_2_, the C–N vibrations, the Amide I peak, and the C–H deformation vibration/C-O stretching vibrations of PVA, PVP, CS + AA, and PEG, respectively, can be seen. Compared to the individual polymer peaks with M8 peaks, these peaks were aligned with a slight shift in wavelength, indicating that the polymers were successfully blended and interacted with each other. For M8, the peak at 3314.76 cm^−1^ may correspond to the –OH stretching vibration with secondary –NH groups of CS. While the peak is at 1636.28 cm^−1^, the corresponding functional groups can be the C=O stretching vibration of PVP, the O–H bending mode of the –OH groups (due to the high amount of water), the C=O stretching vibration of PEG, or the C=O stretching (Amide I) of CS + AA. The peak at 1280.48 cm^−1^ may correspond to the C-H bond in the pyranose ring of CS + AA. Additionally, the peak at 1080.17 cm^−1^ may correspond to the C-O stretching vibrations or C–O–C symmetric stretching of PEG, or the shift of the free amino group –NH_2_ at the C2 position of glucosamine in CS + AA.

### 3.3. UV-VIS Analysis

As promising polymeric antimicrobial agents, the light reflectance (from 200 to 700 nm) of the polymers should be investigated. Therefore, the M8 sample was measured using UV-VIS spectrometry (Jasco V-730, scan speed 400 nm/min, data interval 1 nm, response 0.24 s, filter exchange step) to measure the reflectance percentage of the material, as shown in Figure S16.

As shown in Figure S16, M8 has less than 58% reflectance in the UV-C range (<280 nm), ranging from 58 to 72% reflectance in the UV-B range (280–315 nm), 72–93% reflectance in the UV-A range (315–400 nm), and at least 85% reflectance in the visible light spectrum. This indicates that the material is highly reflective in visible light, even though M8 is transparent.

### 3.4. Minimum Bactericidal Concentration Analysis

M8 MIC concentration plus three more concentrations (12.5, 25, and 50% polymer, respectively) were plated on MH agar; MBC was established at 12.5%, coincident with MIC. This indicates the strong antimicrobial activity of the compound.

## 4. Conclusions

The results obtained through Pareto charts, main effect plots, and interaction plots highlight the significance of polymer concentration, with an increase leading to improved inhibition percentage and reduced MIC_50_. However, the influence of CS as the primary factor influencing these changes is evident. Moreover, the interaction between CS and PVP with other polymers emerges as a key factor in achieving enhanced antimicrobial effects. These findings contribute to a deeper understanding of the antimicrobial activities of the studied polymers and provide valuable insights for the development of effective antimicrobial formulations. The MIC_50_ value of M1–M16 was at a polymer percentage of 12.5%. At 12.5% polymer percentage, with the limits of [PVA], [PEG], and [PVP] being 0.002–0.004 g/mL and [CS] being 0.001–0.002 g/mL, using the 2-level full factorial method, the Inhibition percentage is represented by a single equation of: %Inhibition_12.5% polymer percentage_ = 174.1-27812 PVA-18561 PVP-25960 PEG-38752 CS + 9263047 PVA*PVP + 10430763 PVA*PEG + 15397157 PVA*CS + 7088313 PVP*PEG + 7841221 PVP*CS + 14228046 PEG*CS-3367292860 PVA*PVP*PEG-5671998721 PVA*PVP*CS-6619041275 PVA*PEG*CS-3917095529 PVP*PEG*CS + 2273661969470 PVA*PVP*PEG*CS. Based on the obtained equation, economically, the most optimized polymer percentage is at 12.5%, and the concentration of PVA, PVP, PEG, and CS should be 0.002, 0.002, 0.002, and 0.001 mg/mL to reach the inhibition percentage of 99.162%, and that would be MIC_50_, which coincides with the MBC.

## Figures and Tables

**Table 1:** Volume of PVA, PVP, PEG, CS, and additional DI in the polymer blends.

	Volume Added (mL)					Total Volume (mL)
Polymers	PVA	PVP	PEG	CS	DI	
M1	2	2	2	2	2	10
M2	1	2	2	1	4	10
M3	2	1	1	1	5	10
M4	1	2	2	2	3	10
M5	2	1	1	2	4	10
M6	2	1	2	1	4	10
M7	1	1	1	2	5	10
M8	1	1	1	1	6	10
M9	1	1	2	2	4	10
M10	2	2	2	1	3	10
M11	2	2	1	2	3	10
M12	1	2	1	2	4	10
M13	1	2	1	1	5	10
M14	1	1	2	1	5	10
M15	2	1	2	2	3	10
M16	2	2	1	1	4	10

**Table 2:** Concentration of PVA, PVP, PEG, and CS in the polymer blends using the volume of individual polymers added from Table 1.

	Concentration (g/mL)				Mass Fraction (%)			
Polymers	PVA	PVP	PEG	CS	PVA	PVP	PEG	CS
M1	0.004	0.004	0.004	0.002	0.39	0.39	0.39	0.20
M2	0.002	0.004	0.004	0.001	0.20	0.40	0.40	0.10
M3	0.004	0.002	0.002	0.001	0.40	0.20	0.20	0.10
M4	0.002	0.004	0.004	0.002	0.20	0.40	0.40	0.20
M5	0.004	0.002	0.002	0.002	0.40	0.20	0.20	0.20
M6	0.004	0.002	0.004	0.001	0.40	0.20	0.40	0.10
M7	0.002	0.002	0.002	0.002	0.20	0.20	0.20	0.20
M8	0.002	0.002	0.002	0.001	0.20	0.20	0.20	0.10
M9	0.002	0.002	0.004	0.002	0.20	0.20	0.40	0.20
M10	0.004	0.004	0.004	0.001	0.39	0.39	0.39	0.10
M11	0.004	0.004	0.002	0.002	0.40	0.40	0.20	0.20
M12	0.002	0.004	0.002	0.002	0.20	0.40	0.20	0.20
M13	0.002	0.004	0.002	0.001	0.20	0.40	0.20	0.10
M14	0.002	0.002	0.004	0.001	0.20	0.20	0.40	0.10
M15	0.004	0.002	0.004	0.002	0.40	0.20	0.40	0.20
M16	0.004	0.004	0.002	0.001	0.40	0.40	0.20	0.10

**Table 3:** (**a**) The relationship between polymer concentration and inhibition percentage at each dilution interval using a 2-level factorial design with polymer percentages of 100%, 50%, and 25%. (**b**) The relationship between polymer concentration and inhibition percentage at each dilution interval using a 2-level factorial design with polymer percentages of 12.5%, 6.25%, and 3.125%. (**c**) The calculated possible inhibition percentage at different polymer percentages with the concentration of each individual polymer.

**(a)**							
	**%_Polymer_**	**100**		**50**		**25**	
	
$\chi^{2}$		5.5 × 10^−6^		3 × 10^−6^		3.38 × 10^−4^	
$c_{0}$		93.46		99.29		201.9	
$c_{1}$		−3803		2836		−36,037	
$c_{2}$		1457		−12,131		−40,514	
$c_{3}$		1128		−1876		−47,805	
$c_{4}$		5186		−4598		−42,477	
$c_{5}$		501,206		2,058,038		13,394,054	
$c_{6}$		487,671		−1,549,183		13,518,231	
$c_{7}$		613,661		154,846		13,497,532	
$c_{8}$		−1,307,463		2,647,700		16,166,648	
$c_{9}$		−1,608,092		6,943,765		13,299,686	
$c_{10}$		−2,781,154		1,296,601		22,375,418	
$c_{11}$		159,862,331		−222,576,831		−5,097,528,902	
$c_{12}$		−103,833,371		−1,507,737,077		−5,015,421,713	
$c_{13}$		309,606,765		456,423,015		−6,441,969,969	
$c_{14}$		1,140,611,976		−1,372,514,719		−7,336,917,692	
$c_{15}$		−208,114,800,733		187,830,797,222		2,462,469,583,911	
**(b)**							
	**%_Polymer_**	**12.5**		**6.25**		**3.125**	
	
$\chi^{2}$		8.15 × 10^−6^		2 × 10^−3^		1 × 10^−5^	
$c_{0}$		174.1		−152.2		−10.76	
$c_{1}$		−27,812		54,806		2998	
$c_{2}$		−18,561		22,999		−2444	
$c_{3}$		−25,960		−13,282		4633	
$c_{4}$		−38,752		163,016		47,425	
$c_{5}$		9,263,047		−9,141,590		6,321,380	
$c_{6}$		10,430,763		−3,826,608		1,303,958	
$c_{7}$		15,397,157		−43,648,307		−10,885,764	
$c_{8}$		7,088,313		7,807,002		1,481,389	
$c_{9}$		7,841,221		−23,647,727		−5,905,741	
$c_{10}$		14,228,046		4,956,960		−12,761,287	
$c_{11}$		−3,367,292,860		11,004,673		−1,925,949,284	
$c_{12}$		−5,671,998,721		9,273,389,515		−1,364,561,504	
$c_{13}$		−6,619,041,275		4,061,533,740		2,646,742,454	
$c_{14}$		−3,917,095,529		−4,602,633,086		1,673,360,195	
$c_{15}$		2,273,661,969,470		−246,416,795,664		213,203,200,954	
**(c)**							
	**%_Polymer_**	**100**	**50**	**25**	**12.5**	**6.25**	**3.125**
	
Highest % Inhibition		93.739	96.872	86.524	99.162	99.229	36.803
[PVA] (mg/mL)		0.002	0.002	0.002	0.002	0.004	0.004
[PVP] (mg/mL)		0.002	0.004	0.002	0.002	0.004	0.004
[PEG] (mg/mL)		0.002	0.004	0.004	0.002	0.004	0.002
[CS] (mg/mL)		0.002	0.002	0.002	0.001	0.002	0.001

**Table 4:** The relationship between polymer concentration and inhibition percentage at different polymer percentages using linear regression.

	%_Polymer_	100	50	25	12.5	6.25	3.125
	
$R^{2}$		29.21	64.36	13.92	76.57	87.8	55.85
$c_{0}$		93.62	87.74	76.39	107.67	−31.6	38.56
$c_{1}$		−361	396	754	−555	7209	860
$c_{2}$		−79	−27	−103	−507	1314	−960
$c_{3}$		−348	−225	173	916	432	−1168
$c_{4}$		357	3844	2379	−10,837	47,936	−8877

**Table 5:** The relationship between polymer blend and MIC_50_ using a 2-level factorial design.

Variables	Values
$\chi^{2}$	5.90203 × 10^−5^
$c_{0}$	52.08
$c_{1}$	−15,625
$c_{2}$	−10,417
$c_{3}$	−4167
$c_{4}$	−25,000
$c_{5}$	4,687,500
$c_{6}$	3,125,000
$c_{7}$	8,333,333
$c_{8}$	1,041,667
$c_{9}$	6,250,000
$c_{10}$	2,083,333
$c_{11}$	−1,041,666,667
$c_{12}$	−2,604,166,667
$c_{13}$	−1,562,500,000
$c_{14}$	−520,833,333
$c_{15}$	520,833,333,333

**Table 6:** The relationship between polymer blend and MIC_50_ using linear regression.

Variables	Values
$R^{2}$	65.28
$c_{0}$	16.93
$c_{1}$	−781
$c_{2}$	−260
$c_{3}$	−260
$c_{4}$	−3125

**Table 7:** FTIR analysis of polymers.

Chemicals	Wavelength (cm^−1^)	Functional Group	References
PVA	3298.61	O–H symmetric stretching	[66,67]
	1635.99	O–H bending mode of the –OH groups	[68,69]
	1274.28	C–H bending vibration of CH_2_	[66,68,70]
PVP	3316.45	O-H symmetric stretching	[71,72,73,74,75]
	1636.67	C=O stretching vibration O–H bending mode of the –OH groups	[71,72,73,74] [68,69]
	1467.79	CH_2_ scissor	[75,76]
	1467.67	CH_2_ scissor	[75,76]
	1426.55	C–H vibration	[73]
	1294.62	C–N vibrations	[71,72,73,74]
PEG	3312.73	O–H symmetric stretching	[77,78,79,80]
	1635.95	C=O stretching vibration O–H bending mode of the –OH groups	[77] [68,69]
	1351.80	C–H deformation vibrations	[77,81]
	1082.85	C–O stretching vibrations C–O–C symmetric stretching	[77,81,82] [78,79,80]
CS + AA	3320.67	O–H symmetric stretching and -NH symmetrical vibration	[67]
	1636.09	C=O stretching (Amide I) C=O stretching vibration	[67,83] [71,72,73,74]
	1394.97	CH_2_ in CH_2_OH group	[84]
	1278.48	C–H bond in pyranose ring	[84]
	1091.42	–C–O– stretching vibration	[67,85]
	1016.12	free amino group –NH_2_ at the C2 position of glucosamine	[84]
M8	3314.76	–OH stretching vibration of PVA, PVP, PEG with secondary -NH groups of CS + AA	This research
	1636.28	C=O stretching vibration of PVP, or O–H bending mode of the –OH groups (due to the high amount of water), or C=O stretching vibration of PEG, or C=O stretching (Amide I) of CS + AA	This research
	1280.48	C–H bond in pyranose ring of CS + AA	This research
	1080.17	C–O stretching vibrations or C–O–C symmetric stretching of PEG free amino group –NH_2_ at the C2 position of glucosamine in CS + AA	This research

## Data Availability

All data that supports the findings of this study are included within the article (and any Supplementary Files).

## Supplementary Materials

The following supporting information can be downloaded at: https://www.mdpi.com/article/10.3390/pharmaceutics15102453/s1, Figure S1: Inhibition percentage v. polymers percentage. Figure S2: The Pareto chart of the effects of M1–M16 on MIC_50_. Figure S3: The Pareto chart of the effects of M1–M16 on inhibition percentage when the polymer blends with polymer percentage of (a) 100%, (b) 50%, (c) 25%, (d) 12.5%, (e) 6.25%, (f) 3.125%. Figure S4: Main effect plots for means on MIC_50_ for M1-M16 using 2-level factorial design methods. Figure S5: Main effect plots for means on inhibition percentage for M1–M16 polymer percentage of (a) 100%, (b) 50%, (c) 25%, (d) 12.5%, (e) 6.25%, (f) 3.125% using by 2-level factorial design methods (left to right). Figure S6: Main effect plots on MIC_50_ for M1–M16 using by (a) Taguchi methods main effect plots for means, (b) Linear regression. Figure S7: Main effect plots for S/N ratios on inhibition percentage for M1- polymer percentage of (a) 100%, (b) 50%, (c) 25%, (d) 12.5%, (e) 6.25%, (f) 3.125% using Taguchi methods (left to right. Figure S8: Interaction plots for means on MIC_50_ for M1-M16 using 2-level factorial design methods. Figure S9: Interaction plots for means on inhibition percentage for M1–M16 with polymer percentage of (a) 100%, (b) 50%, (c) 25%, (d) 12.5%, (e) 6.25%, (f) 3.125% using by 2-level factorial design methods (left to right). Figure S10: Interaction plots for means on MIC_50_ for M1-M16 using Taguchi methods for S/N ratios. Figure S11: Interaction plots for S/N ratios on inhibition percentage for M1–M16 with polymer percentage of (a) 100%, (b) 50%, (c) 25%, (d) 12.5%, (e) 6.25%, (f) 3.125% using by Taguchi methods (left to right). Figure S12: The relationship between inhibition percentage and polymers concentration with experimental fittings. Figure S13: Residual plots of the linear regression of polymer blend concentration and inhibition percentage at different polymers percentage (a) 100%, (b) 50%, (c) 25%, (d) 12.5%, (e) 6.25%, (f) 3.125% (left to right). Figure S14: Residual plots of the linear regression of polymer blend concentration and MIC_50_. Figure S15: FTIR analysis of polymers. Figure S16: Reflectance percentage of M8 from 200 nm to 700 nm.
