# Supplementary material for: Relationship between the Polymer Blend Using Chitosan, Polyethylene Glycol, Polyvinyl Alcohol, Polyvinylpyrrolidone, and Antimicrobial Activities against Staphylococcus aureus
Source: Pharmaceutics. 2023 Oct 13;15(10):2453. doi: 10.3390/pharmaceutics15102453 (PMC10610092; doi:10.3390/pharmaceutics15102453)
Supplement: Supplementary file 1 [file pharmaceutics-15-02453-s001.zip › pharmaceutics-2622032-supplementary.pdf]

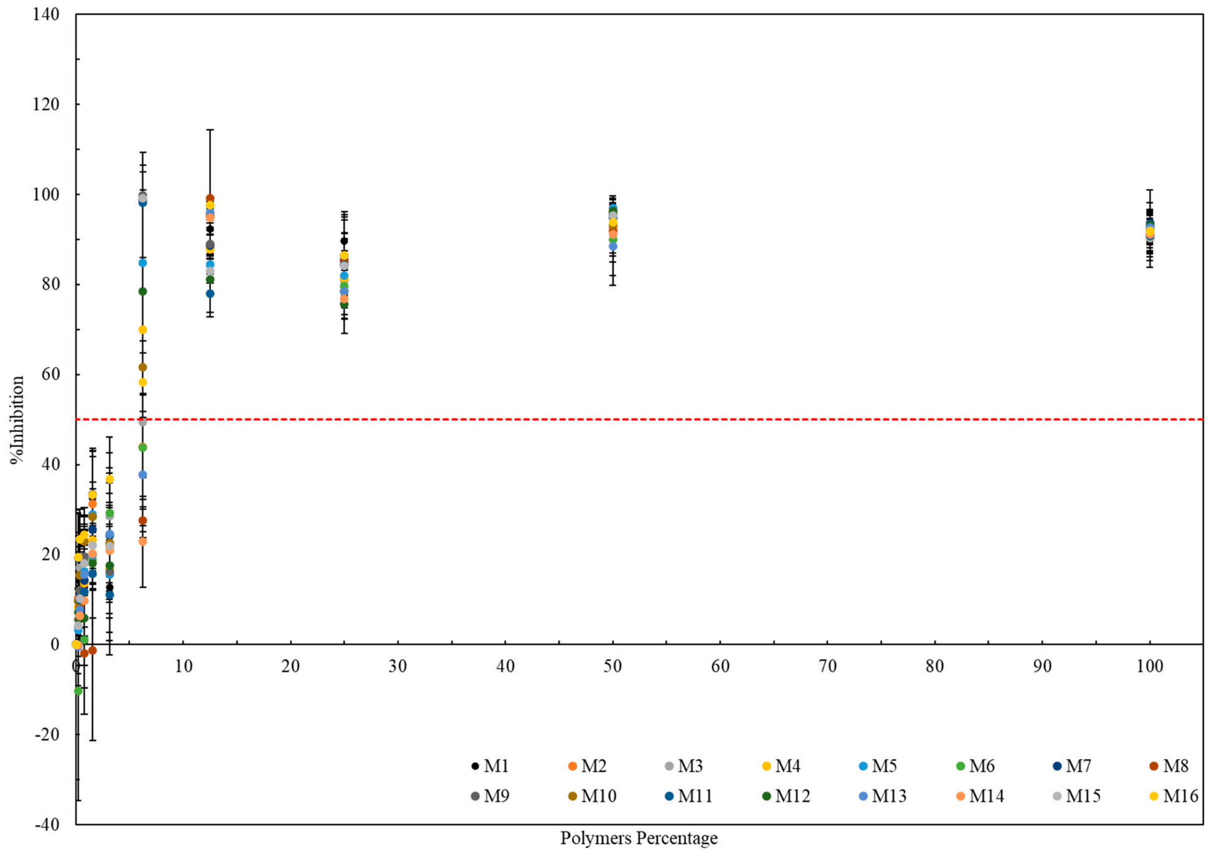

Figure S1. Inhibition percentage v. polymers percentage.

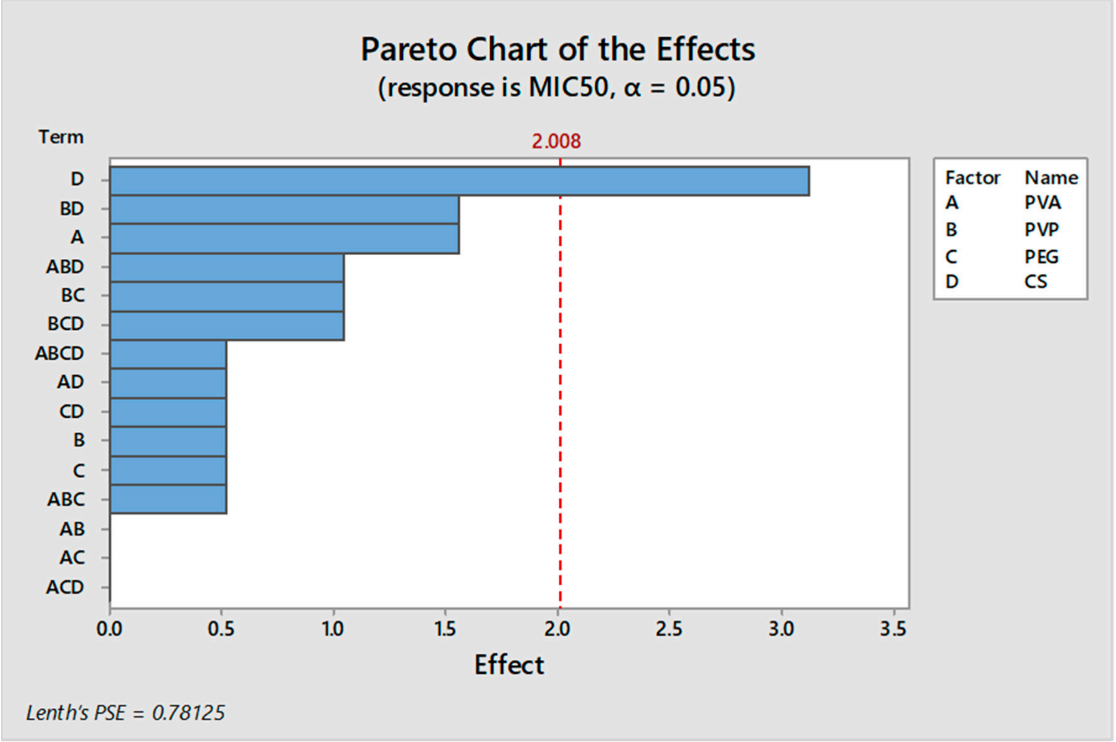

Figure S2. The Pareto chart of the effects of M1-M16 on MIC_{50}.

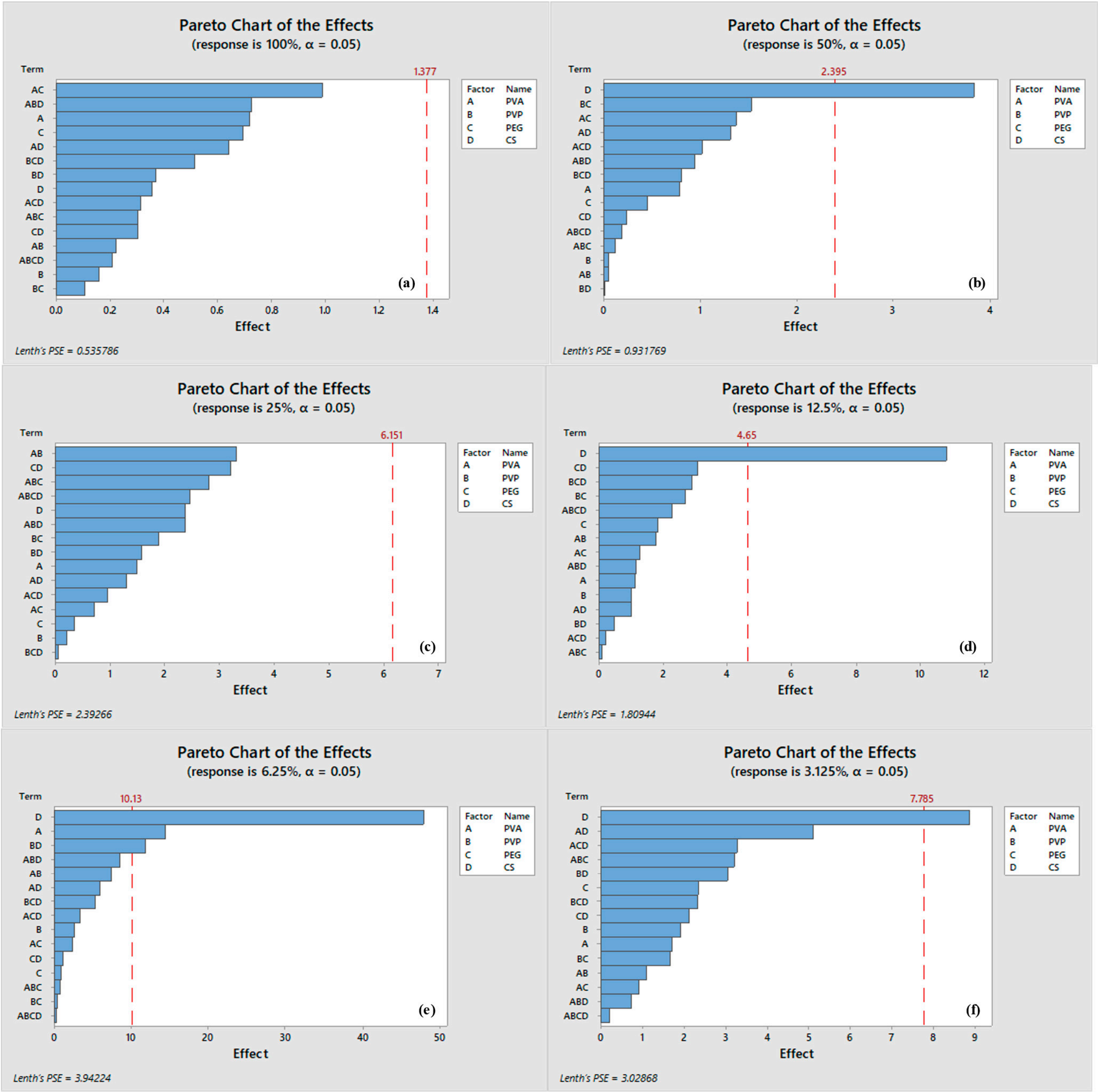

Figure S3. The Pareto chart of the effects of M1-M16 on inhibition percentage when the polymer blends with polymer percentage of (a) 100%, (b) 50%, (c) 25%, (d) 12.5%, (e) 6.25%, (f) 3.125%.

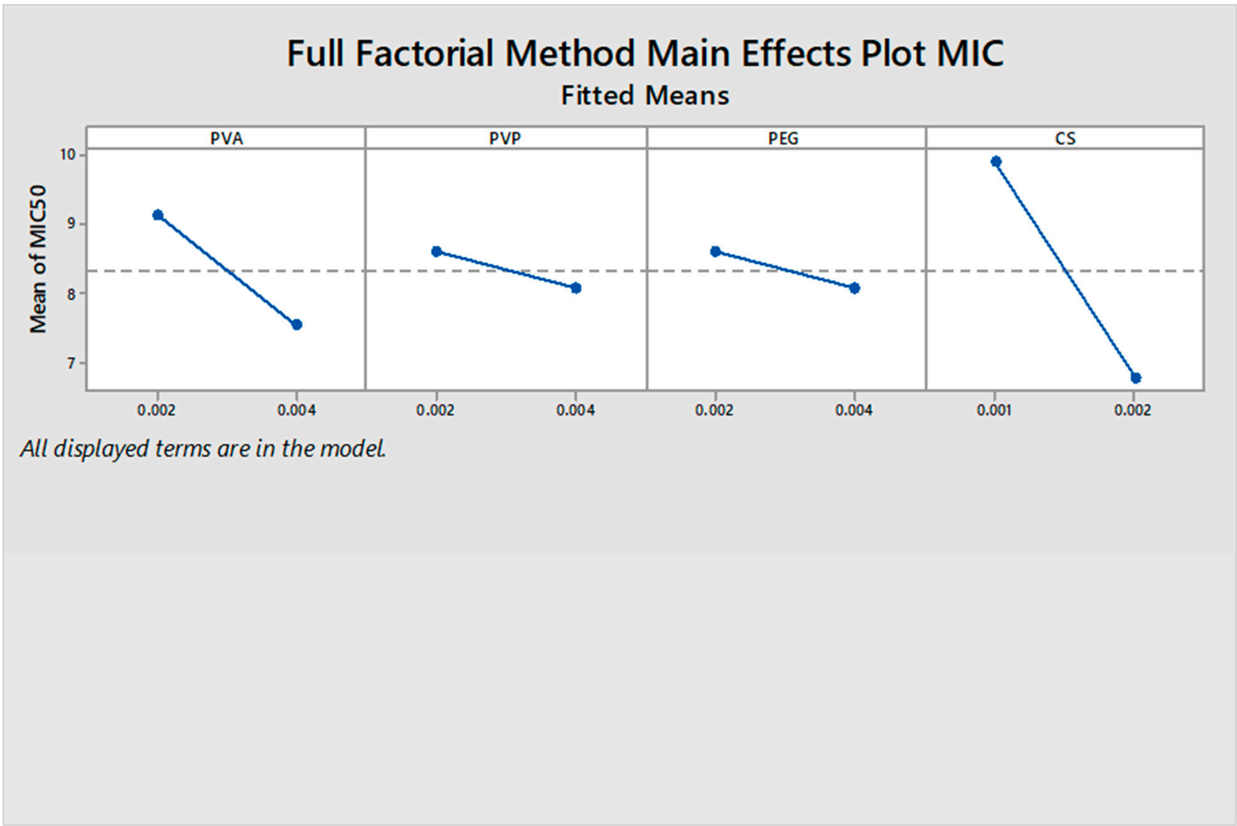

Figure S4. Main effect plots for means on MIC_{50} for M1-M16 using 2-level factorial design methods.

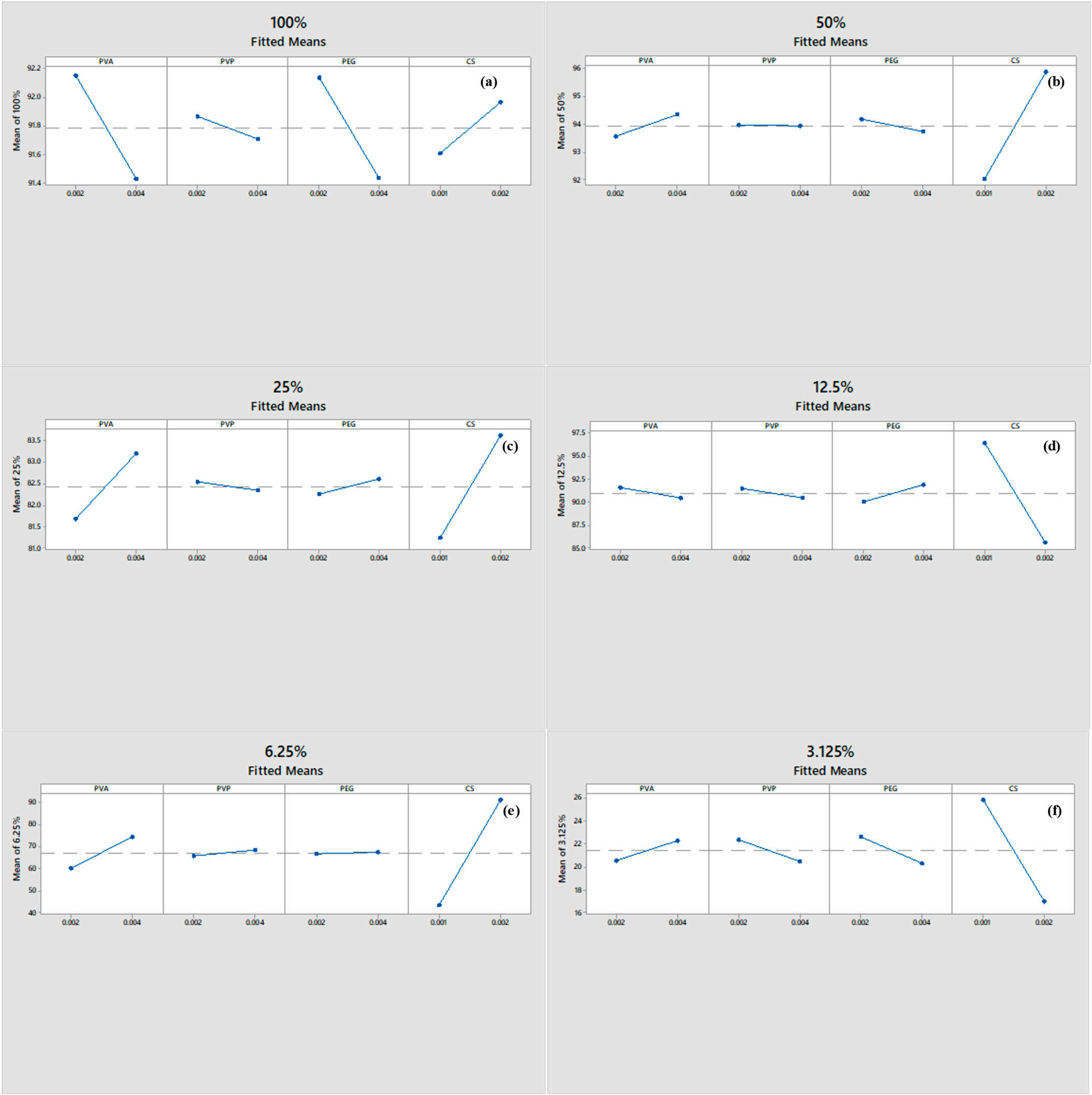

Figure S5. Main effect plots for means on inhibition percentage for M1-M16 polymer percentage of (a) 100%, (b) 50%, (c) 25%, (d) 12.5%, (e) 6.25%, (f) 3.125% using by 2-level factorial design methods (left to right).

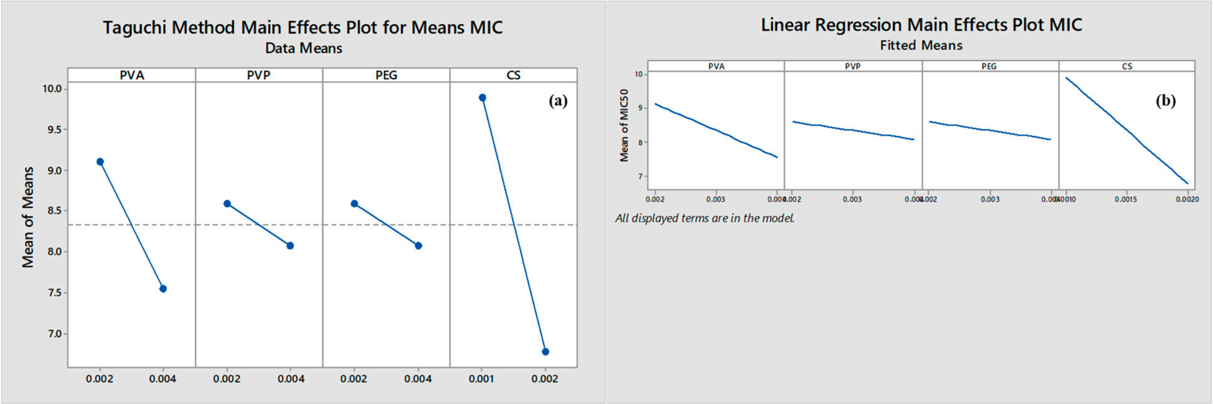

Figure S6. Main effect plots on MIC_{50} for M1-M16 using by (a) Taguchi methods main effect plots for means, (b) Linear regression.

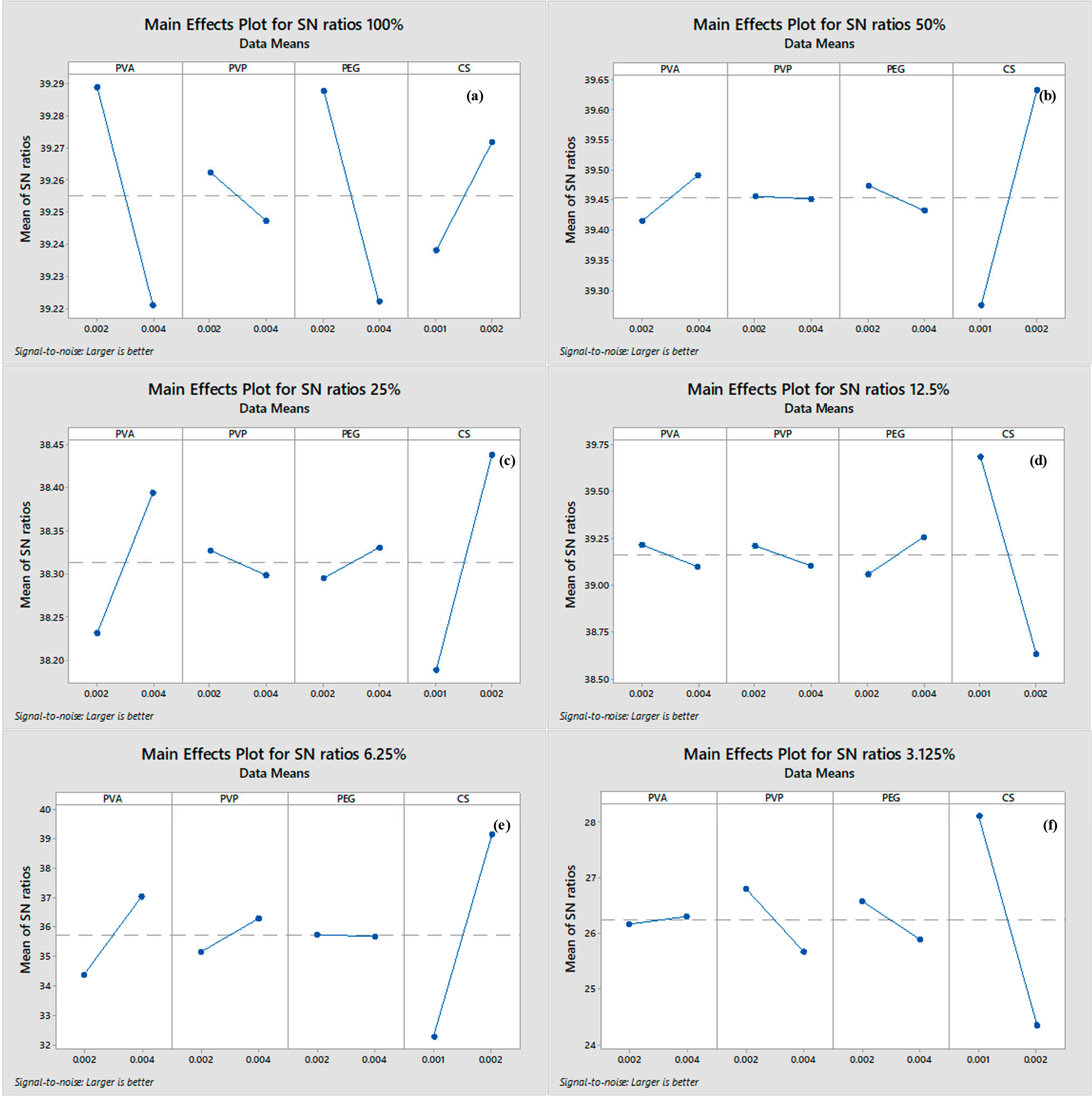

Figure S7. Main effect plots for S/N ratios on inhibition percentage for M1- polymer percentage of (a) 100%, (b) 50%, (c) 25%, (d) 12.5%, (e) 6.25%, (f) 3.125% using Taguchi methods (left to right).

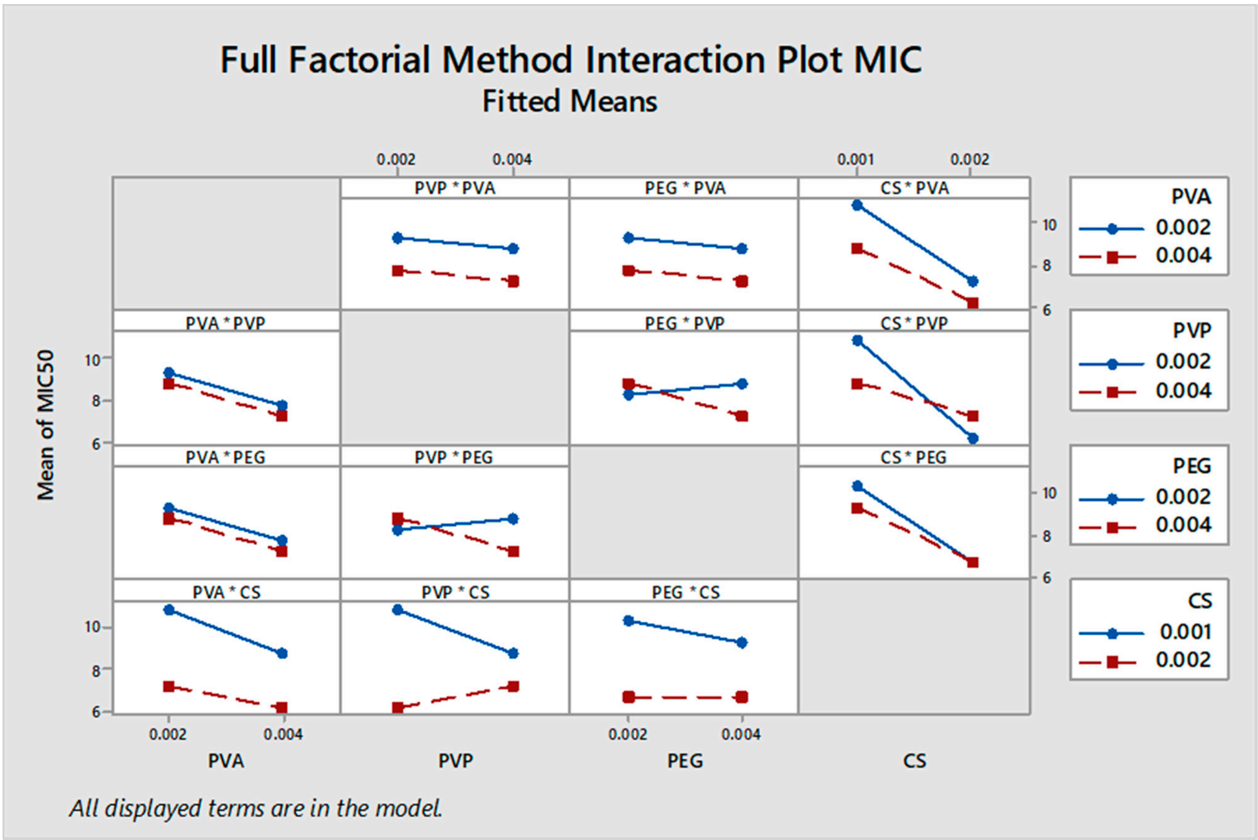

Figure S8. Interaction plots for means on MIC_{50} for M1-M16 using 2-level factorial design methods.

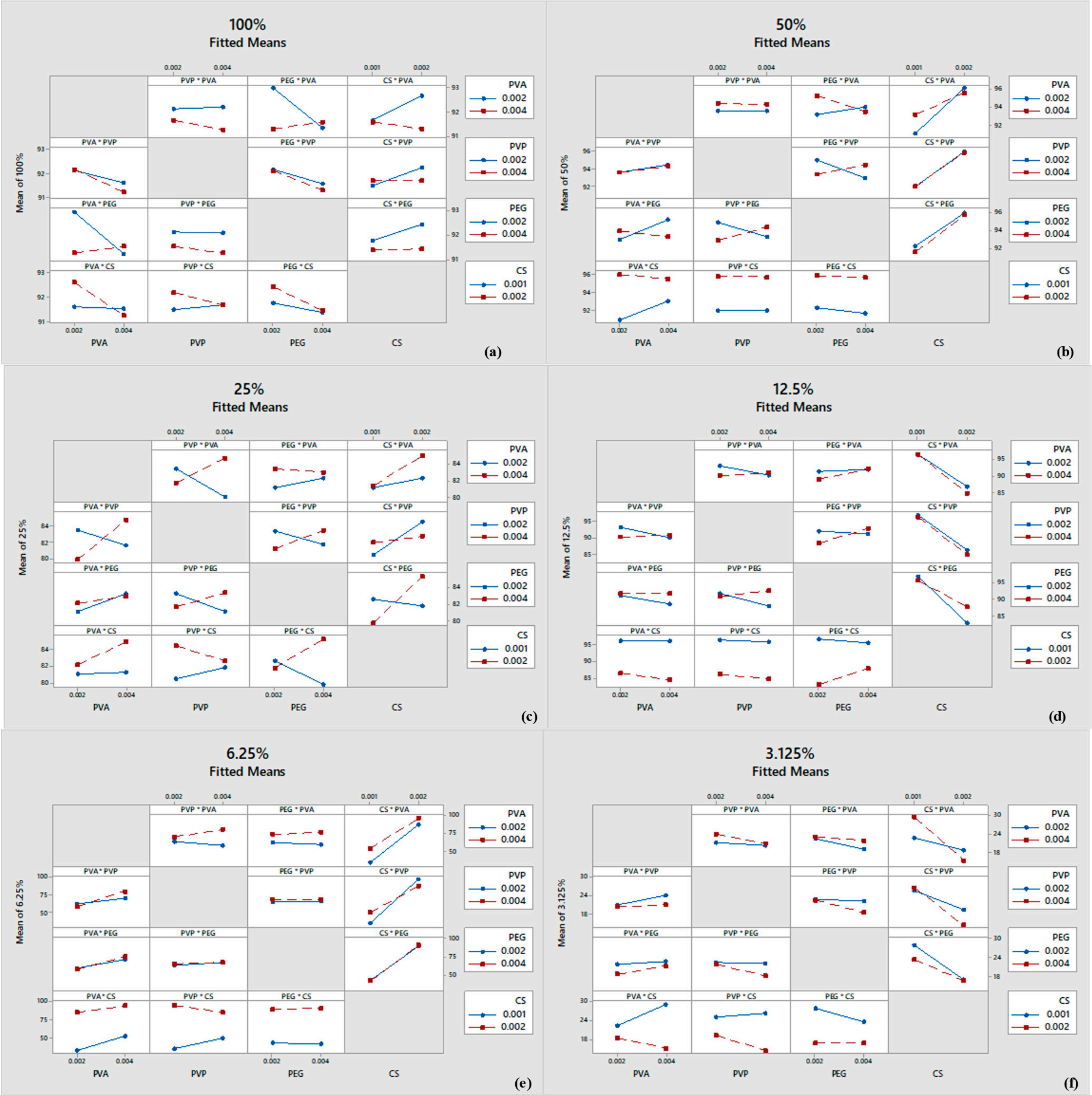

Figure S9. Interaction plots for means on inhibition percentage for M1- M16 with polymer percentage of (a) 100%, (b) 50%, (c) 25%, (d) 12.5%, (e) 6.25%, (f) 3.125% using by 2-level factorial design methods (left to right).

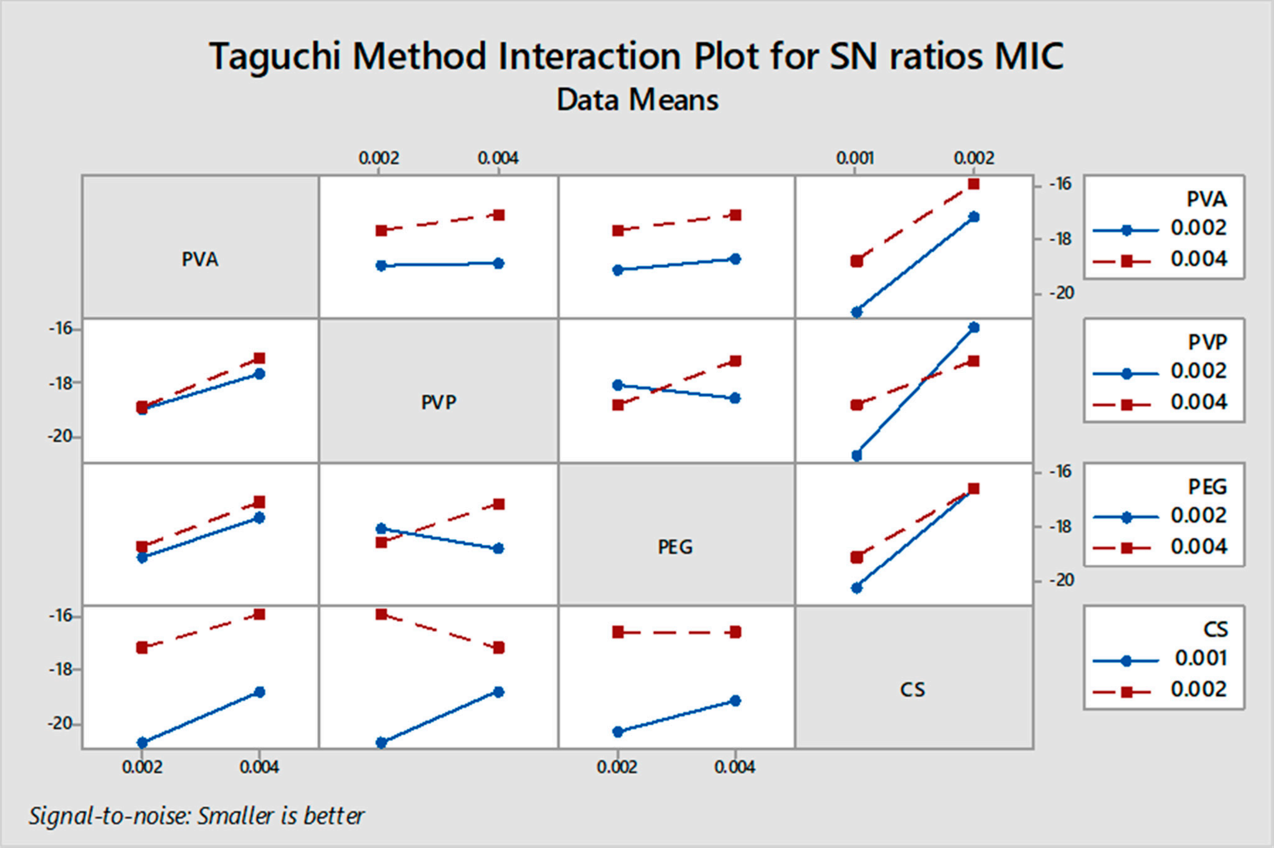

Figure S10. Interaction plots for means on MIC_{50} for M1-M16 using Taguchi methods for S/N ratios.

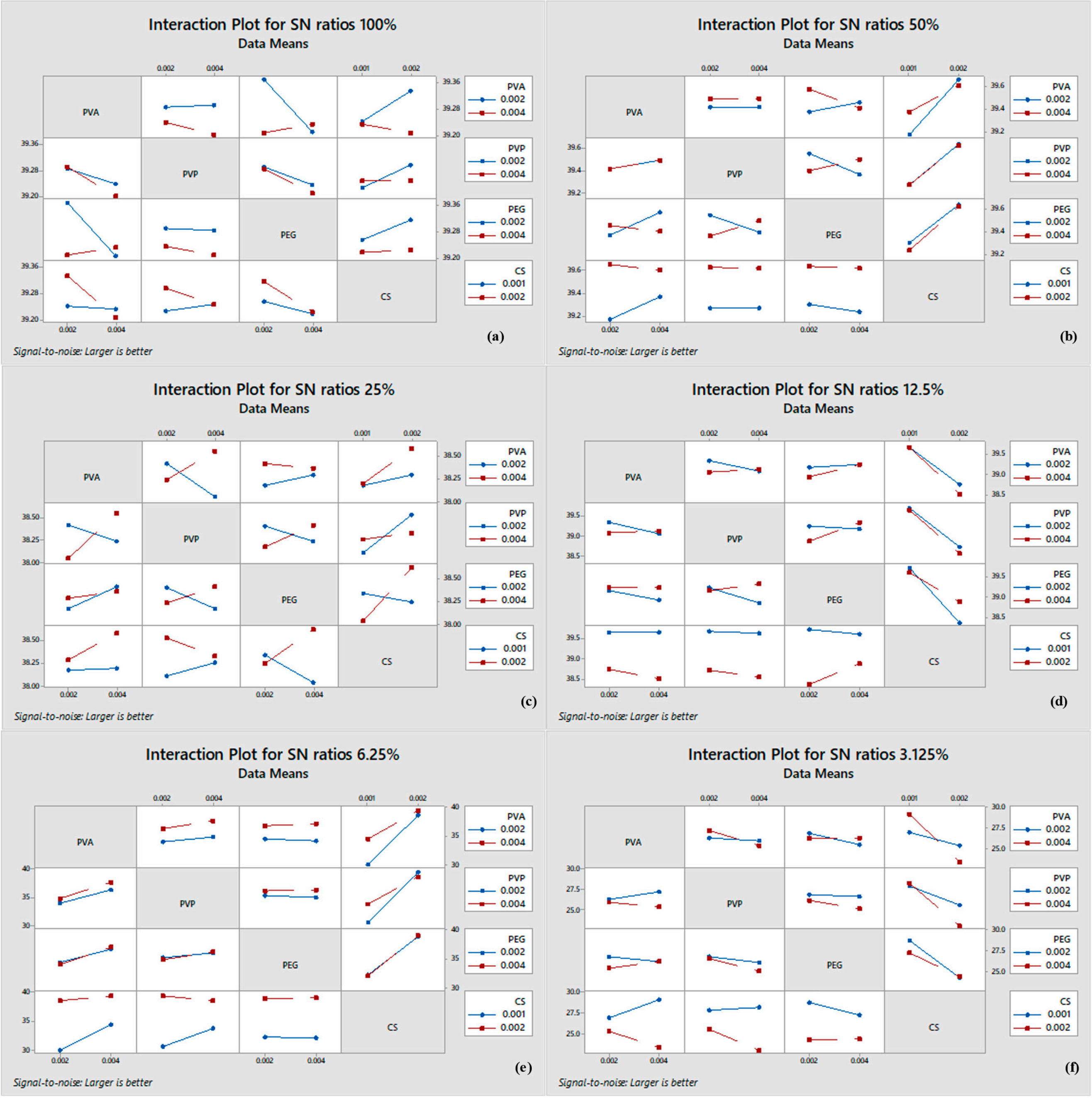

Figure S11. Interaction plots for S/N ratios on inhibition percentage for M1-M16 with polymer percentage of (a) 100%, (b) 50%, (c) 25%, (d) 12.5%, (e) 6.25%, (f) 3.125% using by Taguchi methods (left to right).

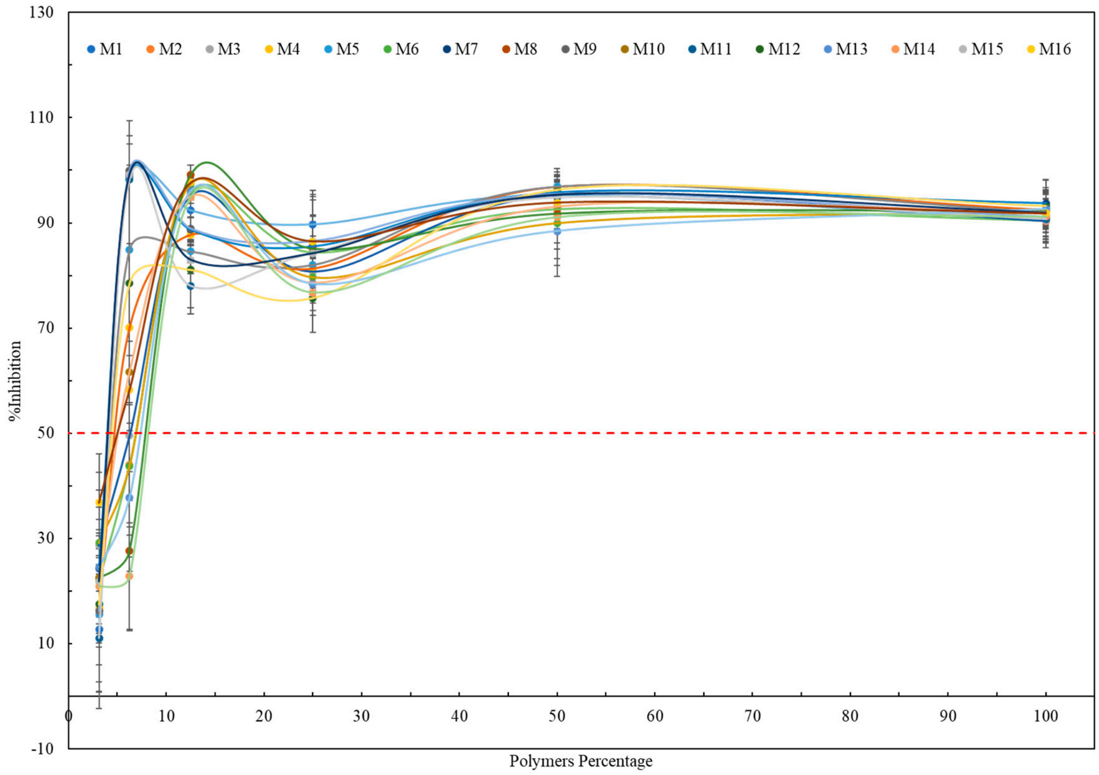

Figure S12. The relationship between inhibition percentage and polymers concentration with experimental fittings.

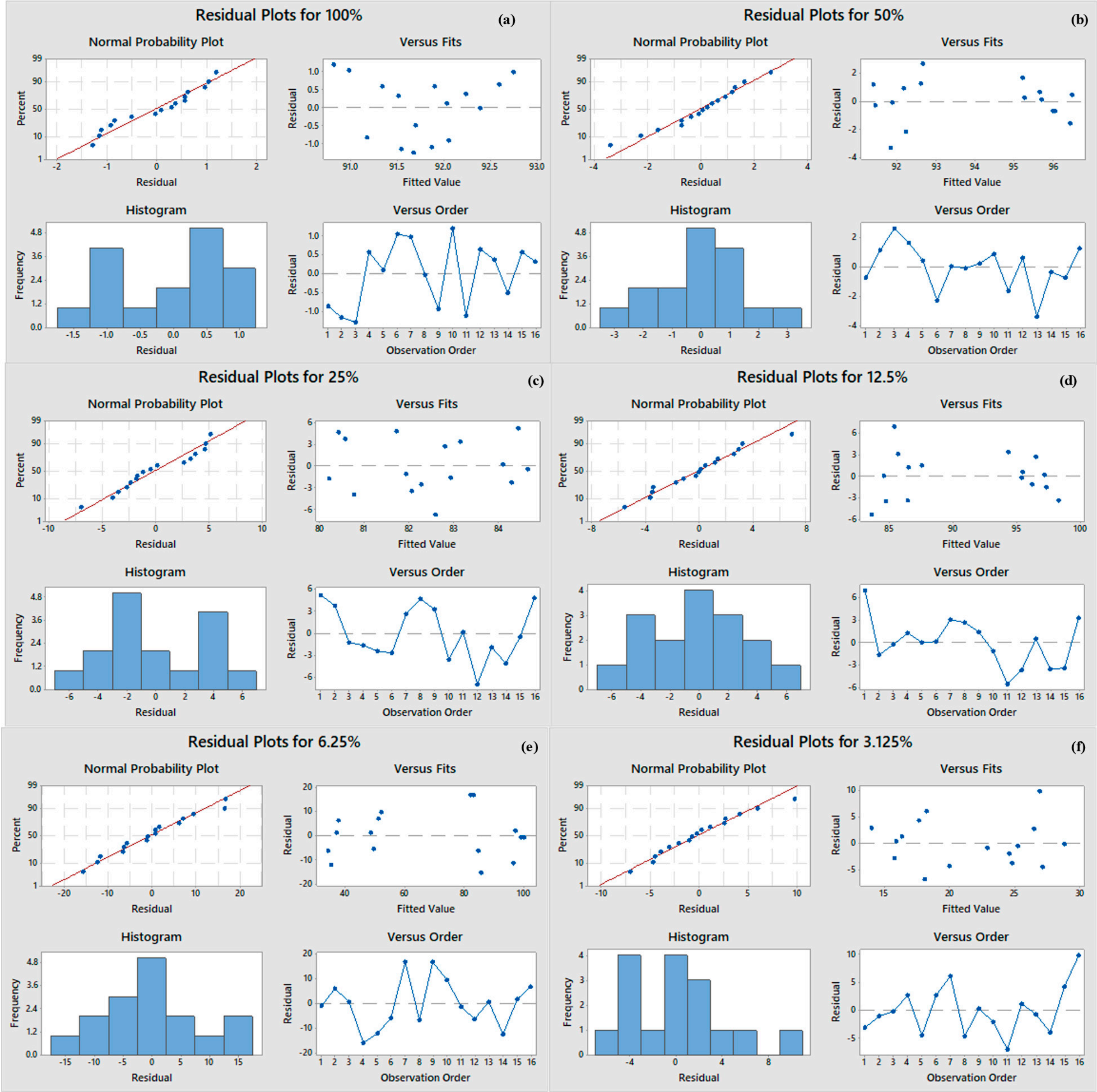

Figure S13. Residual plots of the linear regression of polymer blend concentration and inhibition percentage at different polymers percentage (a) 100%, (b) 50%, (c) 25%, (d) 12.5%, (e) 6.25%, (f) 3.125% (left to right).

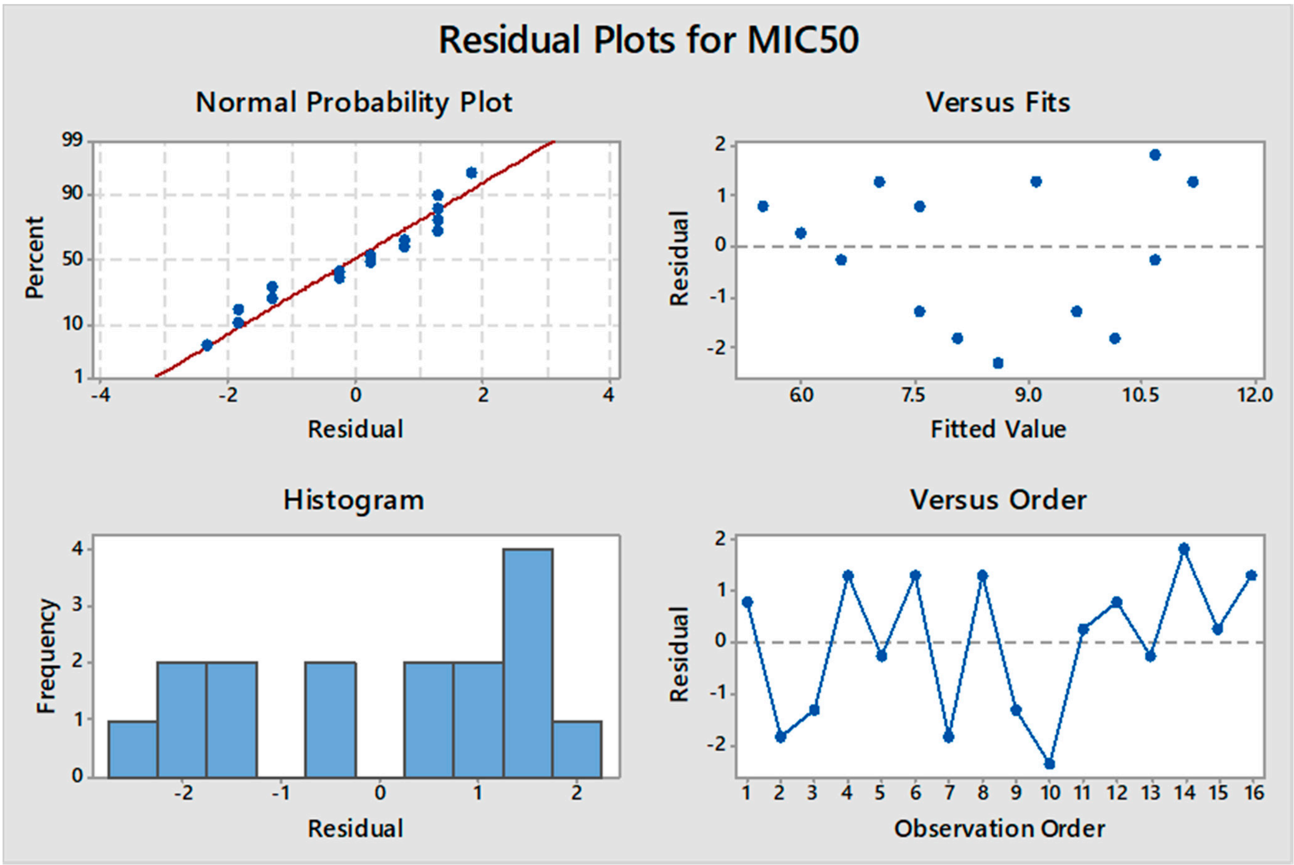

Figure S14. Residual plots of the linear regression of polymer blend concentration and MIC_{50}.

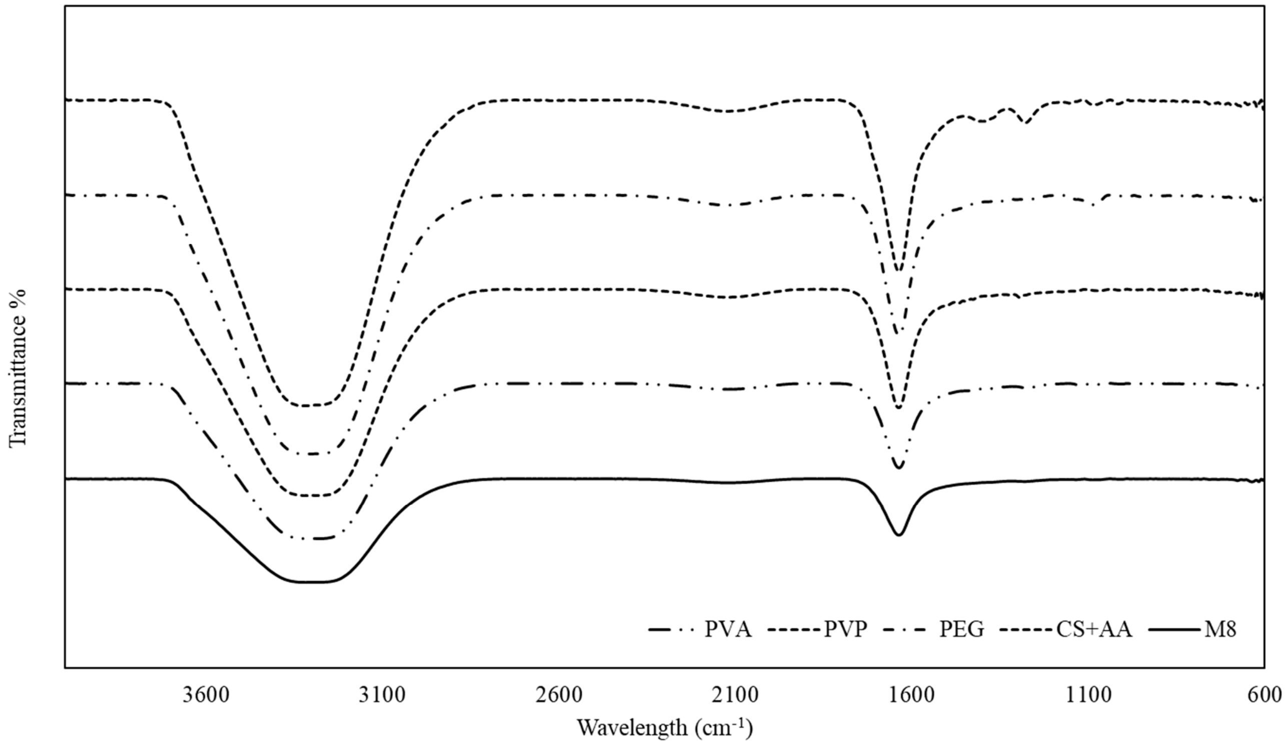

Figure S15. FTIR analysis of polymers.

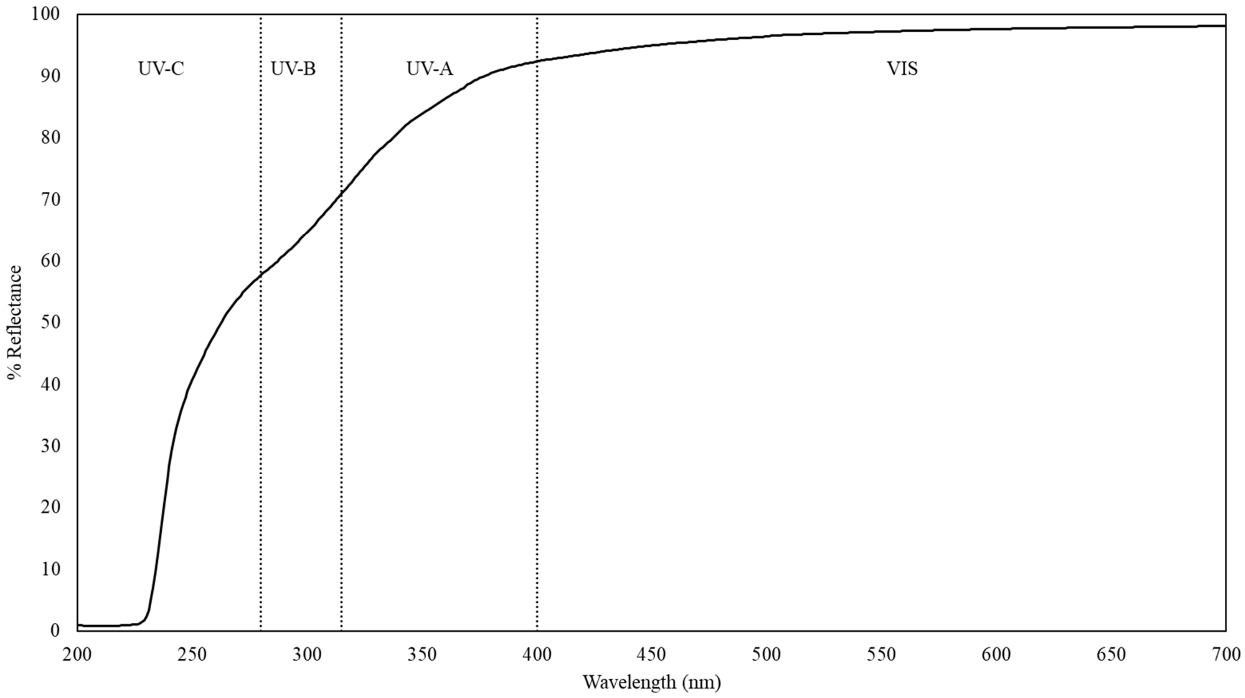

Figure S16. Reflectance percentage of M8 from 200 nm to 700 nm.
